# Weight Bias Internalization Is Inversely Associated with Adherence to the Mediterranean Diet: The Greek Lifestyle and Obesity-Related Bias Survey

**DOI:** 10.3390/nu18050866

**Published:** 2026-03-07

**Authors:** Maria Dimitriou, Natalia Chatzaki, Dimitra Kostara, Maria-Eleni Tsialta, Alexandra Miliou, Sofia Mpanti, Lydia Stalidi, Maria G. Grammatikopoulou, Dimitrios Poulimeneas

**Affiliations:** 1 Department of Nutritional Science and Dietetics, School of Health Sciences, University of the Peloponnese, Antikalamos, GR-24100 Kalamata, Greece; m.dimitriou@go.uop.gr (M.D.);; 2 Immunonutrition Unit, Department of Rheumatology and Clinical Immunology, Faculty of Medicine, School of Health Sciences, University of Thessaly, Biopolis, GR-41223 Larissa, Greece

**Keywords:** dietary assessment, diet quality, dietary intake, habitual intake, overweight, weight bias, weight stigma

## Abstract

**Background/Objectives**: Internalized weight bias has been linked to adverse mental health outcomes and maladaptive eating-related behaviors. However, its relationship with habitual dietary intake and overall diet quality remains insufficiently explored. The objective of this study was to examine associations between internalized weight bias and habitual energy intake, macronutrient composition, and adherence to the Mediterranean diet among adults with a history of overweight or obesity. **Methods**: In this web-based cross-sectional study, 484 adults with a history of excess body mass index completed validated assessments of internalized weight bias (Weight Bias Internalization Scale–Modified; WBIS-M) and usual dietary intake (69-item Food Frequency Questionnaire). Adherence to the Mediterranean diet (MeDi) was assessed via the MedDietScore. Multivariable analyses adjusted for several covariates were performed. **Results**: Higher levels of internalized weight bias were associated with lower adherence to the MeDi (B_adj_ = −0.670, *p* = 0.025). Higher adherence to the MeDi was associated with reduced odds of being classified in the medium or the highest WBIS-M tertile, corresponding to an approximately 5% reduction per 1-unit increment in the MedDietScore. No associations were observed between internalized weight bias and total energy intake. At the macronutrient level, higher internalized weight bias was associated with higher saturated fat intake, independent of total energy intake. **Conclusions**: Internalized weight bias was associated with poorer habitual diet quality and unfavorable macronutrient profiles, independent of total energy intake. These findings suggest that internalized weight bias relates to qualitative differences in habitual food choices, highlighting the potential importance of addressing weight bias in efforts to improve diet quality among adults with overweight or obesity.

## 1. Introduction

People with overweight and obesity are known to exhibit less favorable dietary patterns compared with individuals of lower body weight. Evidence from observational studies indicates lower overall diet quality, higher consumption of energy-dense and ultra-processed foods, lower intake of fruits, vegetables and whole grains, and poorer adherence to dietary guidelines among populations with excess body weight [1,2]. These dietary patterns are strongly associated with increased cardiometabolic risk, including type 2 diabetes, cardiovascular disease, and all-cause mortality, and contribute to the development and persistence of obesity-related chronic diseases [3,4]. Despite extensive public health efforts promoting healthier diets—such as dietary guidelines and culturally relevant models like the Mediterranean diet (MeDi)—improvements in diet quality among individuals with overweight and obesity remain limited, underscoring the complexity of dietary behavior change in this population [5,6].

The persistence of suboptimal dietary patterns highlights the need to better understand the barriers that hinder the adoption and maintenance of healthier eating behaviors among individuals with excess body weight. Structural and environmental factors, including food affordability, access, and time-related constraints, are frequently reported as key contextual barriers within the weight-management context [7,8]. Individual-level factors such as stress, emotional distress, and competing life demands may further impede sustained dietary change [7,9,10]. However, these factors alone do not fully explain the heterogeneity in dietary behaviors observed among individuals exposed to similar environments, suggesting that psychosocial barriers also play a critical and unexplored role.

Dietary behaviors may be conceptualized as the result of interacting environmental and psychosocial factors [11]. Within this context, weight-related psychological experiences may represent important yet insufficiently explored determinants of habitual dietary intake. Internalized weight bias has emerged as a potent psychosocial barrier to healthy eating among individuals with overweight and obesity. Internalized weight bias refers to the extent to which individuals endorse and apply negative societal stereotypes about body weight to themselves, leading to feelings of shame, self-blame, and diminished self-worth [12]. A growing body of literature has linked internalized weight bias to adverse mental health outcomes and maladaptive eating-related behaviors, including emotional eating, binge eating, disinhibited eating, rigid dietary restraint, and lower intuitive eating [13,14,15]. These behaviors may represent coping responses to stigma-related distress. Moreover, when sustained over time, these responses may undermine dietary quality and adherence to healthy dietary recommendations.

Despite increasing recognition of its clinical and public health relevance, weight bias internalization remains understudied in relation to dietary intake per se. Experimental laboratory studies demonstrate that exposure to weight stigma can acutely increase energy intake (in the form of highly palatable snacks) [16]. Nevertheless, these findings are limited to short-term effects, and there is currently no evidence demonstrating sustained or long-term impacts of weight stigma on habitual energy intake. Observational studies provide some indication that stigma is associated with unfavorable diet-related behaviors, including breakfast skipping, greater consumption of fast food and sugar-sweetened beverages, and irregular meal patterns [17,18]. In clinical contexts, among metabolic surgery patients, higher levels of experienced or internalized weight stigma have been associated with poorer adherence to post-surgical dietary recommendations [19]. Nonetheless, to the best of our knowledge, no studies have assessed habitual dietary intake and quality using validated dietary assessment tools.

To address this gap, the present study aimed to examine the relationship between internalized weight bias and habitual dietary intake among adults with a history of overweight or obesity. Specifically, we investigated whether internalized weight bias was associated with energy and macronutrient intake, as well as adherence to the MeDi.

## 2. Materials and Methods

### 2.1. Study Description

The Greek Lifestyle and Obesity-Related Bias Survey is a cross-sectional study, with the primary aim to delineate lifestyle correlates of weight bias internalization among Greek adults aged 18–65 years old with current or previous excess body weight. The survey is delivered online, with data collected and managed using REDCap electronic data capture tools, version 14.0.19, developed by the Vanderbilt University (Nashville, TN, USA) and hosted at the University of the Peloponnese (Kalamata, Greece) [20,21]. Participation in the study is voluntary, and subject to specific inclusion and exclusion criteria. All adults aged 18–65 years old, with a history of adult overweight/obesity (i.e., maximum adult BMI ≥ 25 kg/m^2^), regardless of current BMI, were eligible to enroll. Participants presenting with a current BMI within the normal range at the time of assessment were considered individuals with prior excess body weight (i.e., weight-reduced or post-obesity status) [22]. Further inclusion criteria included provision of electronic informed consent. Women currently (or within the previous year) pregnant were excluded from sampling. We followed a convenience sampling technique, through promotion of the study through social media, patient advocacy groups, Dietitian networks, and the University of the Peloponnese newsletter and mailing list. Recruitment was performed in waves (first wave, May–July 2024; second wave, November 2024–April 2025; and the third wave was initiated during November 2025, with expected closure date in June 2026).

### 2.2. Questionnaires and Measures

#### 2.2.1. Sociodemographics

Questions included age (in years), sex (male/female), educational status (primary or secondary education/Bachelor’s/Master’s/Doctorate), family status (single/in a relationship—non-married/married or cohabitating/married–separated/divorced/widowed), occupational status (public sector/private sector/freelancer/student/pensioner/work without pay (i.e., in family business)/unemployed), economic status (5-point Likert-scale, assessing the relationship between income and expenses, ranging from income being much lower than expenses to income being much higher than expenses), and area of residence (large city/smaller city or town/semi-rural area/rural area).

#### 2.2.2. Weight History and Smoking Status

Weight history included questions regarding current anthropometry (weight and height), and minimum, maximum and desired weight in adult life. Furthermore, participants were asked if they had tried to reduce their weight in the previous year. In the case of affirmative responses, participants were asked about the weight loss method (diet, physical activity, drug therapy, metabolic surgery or any combination of the above), and how they achieved weight loss (with the assistance of a Dietitian/Medical Doctor/other professional/with assistance from friends and/or family/by myself). Smoking status was assessed with binary questions regarding current smoking and/or vaping and smoking cessation.

#### 2.2.3. Dietary Intake and Adherence to the Mediterranean Dietary Pattern

Habitual dietary intake of the previous month was assessed with a validated 69-item Food Frequency Questionnaire (FFQ) capturing the major food groups of the typical Greek diet [23]. The FFQ captures the frequency of each item’s consumption (6 possible unique responses, ranging from never to ≥2 times daily), with each item having been assigned a prespecified portion size (i.e., quantity in g or mL). Frequency responses are then multiplied by portion sizes, thus allowing for quantification of habitual intake, based on the instrument’s guidance. Intake of each item was then converted to energy intake (EI) (kcal/day) and macronutrient intake (g of macronutrients, then converted to % of EI), using the same food composition tables used in the validation of the FFQ (i.e., the United States Department of Agriculture [24] or the National Food Composition Tables [25]). The FFQ has shown adequate validity and repeatability in Greek adults of various BMI ranges. Approval of use of the FFQ was granted by the creators of the questionnaire, after personal communication with the principal investigator. The full description of the FFQ (the included food groups and guidance on its analysis) can be found in the original paper describing its design [23].

The MedDietScore was used as a measure of adherence to the Mediterranean dietary pattern. The MedDietScore is a composite index calculated based on the intake of 11 core food groups of the Mediterranean diet (MeDi). Individual ratings range from 1 to 5, with higher scores indicating higher intake of non-refined grains, potatoes, fruits, vegetables, fish and fisheries, and olive oil. A reverse scoring system is assigned to the intake of poultry, red meat, and full-fat dairy. For alcohol intake, a score of 5 was assigned for consumption of more than zero but <300 mL of alcohol/d. A score of 0 was assigned for zero alcohol intake or for ≥700 mL/d. Scores of 4–1 were assigned for the consumption of 600–700, 500–600, 400–500 and 300–400 mL/d (100 mL has 12 g of ethanol concentration), respectively. After summing individual ratings, the MedDietScore is computed, with a range of 0–55, with higher values indicating greater adherence to the MeDi [26].

#### 2.2.4. Weight Bias Internalization

To assess the degree to which participants internalized stereotypes relevant to their weight status, the Greek validated version of the Modified Weight Bias Internalization Scale (WBIS-M) was used [12,27]. The WBIS-M has 11 items, rated on a 7-point Likert scale (1, strongly disagree, to 7, strongly agree). The total WBIS-M score occurs after dividing the sum of each item rating by 11, with the total score ranging from 1 to 7 (higher scores indicating higher internalization of weight-related bias).

#### 2.2.5. Physical Activity Levels and Estimation of Energy Requirements

Physical activity of the previous week was estimated via the validated International Physical Activity Questionnaire—short form (IPAQ-sf) [28]. The IPAQ-sf captures time spent in vigorous and moderate activities, as well as walking. An appropriate Metabolic Equivalent of Task (MET) for each activity was assigned according to the IPAQ-sf guidance (i.e., 8.0 for vigorous activities, 4.0 for moderate and 3.3. for walking) [29]. Sleep duration was collected via the study’s questionnaire and was assigned a MET of 0.95 [30]. The duration of light and sedentary activities was computed by subtracting the daily duration (in hours) of vigorous activities, moderate activities, walking and sleeping from 24; light and sedentary activities were assigned an MET of 1.4 [29]. Physical Activity Level (PAL) was computed after dividing the sum of total MET hours per day by 24.

Estimated Energy Requirements (EERs) were computed following the National Academies for Science, Engineering and Medicine 2023 reference [31]. The report provides sex- and PAL-specific EER equations (validated via the doubly labeled water method), which are suitable for persons across all BMI ranges. The EI to EER ratio (%) was also computed, as a proxy of energy misreporting.

### 2.3. Statistics

Normality of data was explored graphically with Q-Q plots. According to data distribution, continuous variables were presented as mean ± SD (for normally distributed variables), or otherwise as median (Q1, Q3). Differences between continuous variables were examined using independent *t*-test, or the non-parametric equivalent for non-normally distributed data. Differences in categorical variables were examined with X^2^. To provide a better visualization of our data, the sample was divided in tertiles, by WBIS-M score and sex. Differences in continuous variables by tertile were examined with ANOVA (or the equivalent test for non-parametric variables). Regression models were employed to examine the association between WBIS-M score and dietary intake parameters. Covariates were selected a priori based on theoretical relevance and the prior literature suggesting potential associations with both internalized weight bias and dietary intake. Sociodemographic and lifestyle variables were considered potential confounders. However, weight-related variables such as BMI and recent weight control efforts may plausibly lie along the causal pathway linking internalized weight bias with dietary behaviors. Accordingly, sequential models with increasing levels of adjustment were employed to allow evaluation of associations under alternative causal assumptions. Multicollinearity among independent variables was assessed using variance inflation factors (VIFs) and tolerance statistics derived from regression models, including the same covariates as the multinomial analyses. No evidence of problematic multicollinearity was observed (largest VIF values observed = 1.169). Participants with erroneous energy intake (i.e., <600 and >6000 kcal per day) were excluded from analyses. Significance was set at 5%.

## 3. Results

A total of 494 participants provided full responses in all relevant questionnaires; 10 were excluded from the analyses due to reporting erroneous energy intake (one reported EI < 600 kcal, nine reported EI > 6000 kcal). Therefore, the study sample included 484 adults, with their descriptive characteristics (in total and stratified by sex) presented in Table 1. Participants had a median age of 38.0 years and a mean current body mass index (BMI) of 29.7 kg/m^2^; 44.4% were classified as overweight and 34.7% as having obesity. Most participants were highly educated and employed, and the majority resided in urban areas. Although women represent approximately 70% of the sample, significant sex differences were observed only in the cases of family and financial status. Men were more likely to be single, whereas women were more frequently married or in a relationship (*p* < 0.001). Women more often reported income lower than expenses, while men more frequently reported income exceeding expenses (*p* < 0.001). No other statistically significant sex differences were observed.

In the total sample, the mean WBIS-M score was 3.50 ± 1.04, suggesting moderate internalization of weight bias. The WBIS-M presented a weak, negative association with age (rho = −0.203, *p* < 0.001), as well as a weak to modest association with current and maximum BMI (r = 0.397, *p* < 0.001 for both). Compared to men, women had higher weight bias internalization scores (3.63 ± 1.03 vs. 3.18 ± 0.99, *p* < 0.001).

Given the sex differences in weight bias internalization, tertiles of the WBIS-M score by sex were created. Table 2 depicts dietary intake and adherence to the MeDi, in relation to WBIS-M tertiles (by sex). Participants in the highest WBIS-M tertile (compared to the lowest) reported being younger, and having substantially higher current and maximum BMI, as well as a prevalence of either overweight or obesity (*p* < 0.001 for all comparisons). Significantly more individuals in the highest WBIS-M tertile reported weight control efforts during the previous year compared to all other tertiles (*p* = 0.009). Participants in the highest tertile (compared to the lowest) reported lower energy and protein intake (in relation to their body weight). Furthermore, their PAL and EI:EER were also lower, although these differences were not significant. While a trend for higher saturated fat intake in the highest tertile (compared to the lowest) was noted, the only significant difference observed was a lower intake of saturated fat in participants of the lowest tertile compared to the medium (*p* = 0.012). With regard to core MeDi food group intake, compared to those in the highest WBIS-M tertile, participants in the lowest tertile reported higher intake of vegetables (*p* = 0.05), as well as fish and fisheries (*p* = 0.018). A marginal trend for higher non-refined cereal and lower red meat intake was also apparent. In total, participants in the lowest WBIS-M tertile reported higher adherence to the MeDi compared to all other tertiles (Q1 vs. Q2, *p* = 0.001; Q1 vs. Q3, *p* = 0.002).

We then sought to explore the relationship between dietary intake, as well as adherence to the MeDi, with the WBIS-M (both in the raw score, and in WBIS-M tertiles). According to Table 3, an inverse relationship between the WBIS-M and adherence to the MeDi was denoted, with 1-unit increments in WBIS-M scores associated with significant decrements in the MedDietScore (B = −0.670, *p* = 0.025). Relevant to energy and macronutrient intake, while in the crude models WBIS-M score was significantly associated with lower energy and protein intake, these relationships attenuated after adjustment for covariates. In the crude, as well as the fully adjusted model, SFA intake presented a positive association with the WBIS-M.

Each 1-unit increment in the MedDietScore was associated with approximately 5% fewer odds of being classified in the medium or the higher WBIS-M tertile (Table 4). Similarly, higher protein intake was associated with lower odds of being classified in the highest WBIS-M tertile (against the lowest). This finding was not replicated for persons in the medium WBIS-M quartile (compared to the lowest). Greater SFA intake was associated with higher odds of being classified in the medium WBIS-M tertile (against the lowest); no significant association was present for those in the highest WBIS-M tertile. The trend for an inverse association between energy intake and being classified in the highest WBIS-M tertile did not remain significant after adjustment for covariates.

## 4. Discussion

Our results provide insight into the relationship between weight bias and habitual dietary intake among adults with a history of overweight or obesity. Using validated measures of internalized weight bias and usual dietary intake, we observed that higher levels of internalized weight bias were associated with lower adherence to the Mediterranean dietary pattern, whereas no associations were found with total energy intake or indicators of energy misreporting. Collectively, these findings are supportive of an association between internalized weight bias and poorer habitual diet quality, reflecting differences in the types of foods consumed, rather than differences in habitual energy intake.

Previous research has highlighted that individuals with internalized weight bias or exposure to weight stigma engage in behaviors that are recognized indicators of poorer overall dietary quality. Nonetheless, these behaviors have been examined primarily as isolated dietary practices rather than within the context of a broader dietary pattern. For instance, observational studies have linked weight stigma and internalized weight bias to higher consumption of fast food and sugar-sweetened beverages, lower frequency of breakfast consumption, and more irregular eating patterns [32,33]. Other work has reported associations between weight stigma and lower frequency of fruit and vegetable intake, as well as greater reliance on convenience or ultra-processed foods, typically assessed using brief food frequency items or short reference periods [34]. Although these behaviors are well established correlates of lower diet quality, their examination in isolation limits inferences about the overall structure and quality of the habitual diet. While these findings collectively suggest that individuals with internalized weight bias may engage in less healthful eating practices, they do not capture how such behaviors cluster within the overall habitual diet or whether they translate into poorer adherence to a priori dietary patterns. By examining adherence to the Mediterranean diet using a validated assessment of usual intake, the present study extends the literature beyond isolated dietary behaviors and provides evidence that internalized weight bias is associated with lower overall diet quality.

In contrast to its association with dietary quality, internalized weight bias was not associated with energy intake or with indicators of energy misreporting in the present study. This finding is noteworthy given that experimental studies have demonstrated acute effects of exposure to weight-stigmatizing cues on energy intake, particularly from palatable snack foods, in controlled laboratory settings [16,35]. A recent ecological momentary assessment study suggests that experiences of weight stigma may influence eating behavior in the short term, with evidence of greater intake during eating episodes occurring proximal to stigma exposure [36]. However, these effects appear to be context-dependent and temporal, with possible sustained differences in average energy intake remaining unexplored. The absence of an association between internalized weight bias and habitual energy intake in the present study suggests that stigma-related eating responses observed in experimental or momentary contexts may not accumulate into chronic elevations in energy consumption. Relevant to the relationship between weight bias internalization and the energy intake-to-energy requirements ratio, our findings are in line with a pilot study examining predictors of under-reporting among adults with excess body weight, where WBIS-M scores were not associated with energy under-reporting of habitual intake (measured by three 24-h recalls) [37].

At the macronutrient level, protein intake was inversely associated with internalized weight bias across tertiles. At the same time, saturated fat intake was higher among individuals in the medium compared to the lowest tertile. Importantly, these macronutrient differences occurred in the absence of differences in total energy intake, implying selective differences in food choices rather than increased overall food consumption. Lower protein intake and higher saturated fat intake are characteristic of poorer-quality dietary patterns and are consistent with reduced adherence to prudent dietary patterns [38], as well as the MeDi [39]. Furthermore, both protein and saturated fat intake are strongly determined by the types of foods consumed rather than by total energy intake alone. Taken together, the observed combination of stable energy intake with selective differences in macronutrient composition reinforces the interpretation that internalized weight bias is linked to qualitative differences in habitual diet that adversely affect overall diet quality, rather than sustained excess energy intake.

There are several mechanisms that may explain the aforementioned associations of internalized weight bias with poorer diet quality and selective food choices, independent of total energy intake. Internalized weight bias is characterized by chronic feelings of shame, self-blame, and devaluation, which have been consistently linked to elevated psychological distress, depressive symptoms, and reduced self-efficacy [12,15,40]. Nonetheless, internalized weight bias is conceptually different than the psychological conditions mentioned above, as it reflects self-directed stigma rather than global affective disturbances, and has been found to present associations with health behaviors that are independent of depressive symptomatology and self-esteem [12,15]. Such stigma-related distress may be associated with reduced engagement in health-promoting behaviors that require sustained effort, planning, and self-regulation, including the adoption and maintenance of high-quality dietary patterns. In this context, dietary patterns such as the Mediterranean diet—which emphasize regular meal structure, food preparation, and intentional food selection—may be particularly vulnerable to disruption among individuals with higher internalized weight bias. In addition, internalized weight bias has been associated with maladaptive coping strategies, including avoidance-oriented behaviors and disengagement from self-care practices [41,42]. Rather than promoting compensatory overeating per se, stigma-related distress may be associated with a tendency towards more convenient food choices that require fewer cognitive and practical resources to obtain and prepare. Over time, such selective food choices may erode overall diet quality without necessarily affecting habitual energy intake. The majority of our sample reported saturated fat intake exceeding current dietary recommendations, which generally advise limiting it to less than 10% of total energy intake [43]. The positive association identified between internalized weight bias and SFA intake may therefore have clinical relevance, as higher SFA consumption has been consistently linked to increased cardiometabolic risk. These findings suggest that psychosocial factors such as weight bias internalization may be associated with dietary patterns that further deviate from established nutritional recommendations. Finally, internalized weight bias may also interfere with individuals’ relationships with health messaging and dietary guidance. Weight-stigmatizing experiences within healthcare and public health contexts have been shown to reduce trust, motivation, and perceived relevance of dietary recommendations among individuals with higher body weight [44,45]. This disengagement may further hinder adherence to structured dietary models, such as the Mediterranean diet, which are often promoted within clinical and preventive settings. Taken together, these mechanisms suggest that internalized weight bias may be associated with psychosocial processes relevant to engagement in prudent dietary patterns, although the directionality of these relationships cannot be established within the present study design.

Our results must be interpreted in light of the strengths and limitations of the survey. First, to the best of our knowledge, this is the first research item examining the association between internalized weight bias and habitual dietary intake and quality, via the use of validated instruments. Importantly, the inclusion of the energy intake-to-estimated energy requirement ratio (EI:EER) as a covariate strengthens the validity of the findings by accounting for potential bias related to energy misreporting, which is common in populations with overweight and obesity and may be differentially associated with psychosocial factors [46]. The consistency of the observed associations after adjustment for EI:EER increases confidence that the reported relationships reflect true differences in dietary quality and composition rather than artifacts of reporting bias. Given the sensitive nature of weight stigma, the electronic delivery of the survey was deemed preferable, given that electronic, self-administered surveys allow participants to respond in a private and nonjudgmental environment, which may reduce social desirability bias. Limitations stem from the cross-sectional design, which allows solely for reporting associations and not causal inferences, or the possible bidirectionality of dietary behaviors and internalized weight bias. Dietary intake was self-reported, and although validated methods were used and misreporting was addressed analytically, some degree of measurement error is unavoidable. The convenience sample, as well as the online nature of the study, may have introduced selection bias. Furthermore, our sample primarily consisted of women (a common finding in lifestyle and body weight-related surveys [47]), persons with high education and persons living in urban environments, which may hinder the generalizability of our findings in other populations. The inclusion of weight-related variables as possible covariates in the regression models (i.e., BMI and recent weight control efforts)—which may lie along the behavioral pathway linking internalized weight bias with dietary intake—may have introduced partial over-adjustment, potentially attenuating some associations; however, the persistence of the association with saturated fat and diet quality after full adjustment supports the robustness of these findings. Residual confounding by unmeasured psychosocial factors plausibly influencing both dietary behavior and weight bias internalization (i.e., depressive symptomatology and self-esteem measures) cannot be fully excluded.

## 5. Conclusions

The present study provides novel evidence that internalized weight bias is associated with poorer habitual diet quality among adults with a history of overweight or obesity, independent of total energy intake. This observation underscores that stigma-related dietary disparities may not be driven by excess caloric consumption, but rather by psychosocial processes that may influence sustained engagement in healthful eating patterns. From a clinical and public health perspective, these results highlight the importance of addressing internalized weight bias as a potent psychosocial factor to consider in efforts aiming to improve diet quality. Interventions and dietary guidance that fail to consider stigma-related processes may be limited in their effectiveness, even when caloric intake is adequately addressed. Future longitudinal and intervention studies are needed to clarify causal and directional pathways and to determine whether reducing internalized weight bias can support improvements in diet quality and long-term cardiometabolic health.

## Figures and Tables

**Table 1:** Participants’ descriptive characteristics (N = 484).

	Total Sample, N = 484	Women, n = 344	Men, n = 140	*p*
Age (years)	38.0 (23.0, 51.0)	40.0 (23.0, 51.0)	34.0 (23.0, 51.0)	0.384
Weight History				
Current BMI (kg/m^2^)	29.7 ± 6.9	29.7 ± 7.3	29.6 ± 5.8	0.905
Class: Overweight (%)	44.4	43.6	46.5	0.198
Class: Obesity (%)	34.7	34.0	36.4	0.613
Maximum BMI (kg/m^2^)	32.0 ± 7.2	32.1 ± 7.4	31.9 ± 6.5	0.758
Weight, Current: Max (%)	96.0 (88.7, 100)	95.9 (88.6, 100)	96.1 (89.8, 100)	0.782
Weight Control Efforts (% yes)	75.8	**78.8**	**68.6**	**0.012**
Method: Diet (%) ^1^	87.2	88.9	82.3	0.094
Method: Physical activity (%) ^1^	58.3	**52.8**	**74.0**	**<0.001**
Method: Pharmacotherapy (%) ^1^	5.7	7.0	2.1	0.074
Method: Metabolic surgery (%) ^1^	3.0	3.0	3.1	0.932
Education Status (%)				
Primary	1.2	1.2	1.4	0.088
Secondary	16.9	16.3	18.6	
College	20.5	20.6	20.0	
Tertiary	40.7	41.6	38.6	
MSc or PhD	20.7	20.4	21.4	
Family Status (%)				
Single	30.6	**24.7**	**45.0**	**<0.001**
In a Relationship (non-married)	24.0	**26.2**	**18.6**	
Married	38.8	**41.6**	**32.1**	
Divorced	5.2	**6.1**	**2.9**	
Widowed	1.4	**1.5**	**1.4**	
Occupation Status (%)				
Employed	90.7	90.4	91.4	0.618
Pensioner	4.1	3.8	5.0	
Unemployed	5.2	5.8	3.6	
Financial Status (%)				
Income << Expenses	12.4	**15.1**	**5.7**	**<0.001**
Income < Expenses	12.0	**13.4**	**8.6**	
Income = Expenses	39.0	**41.0**	**34.3**	
Income > Expenses	29.3	**25.0**	**40.0**	
Income >> Expenses	7.2	**5.5**	**11.4**	
Residence (%)				
Capital of Prefecture	61.4	60.2	64.3	0.658
Other City	15.7	16.9	12.9	
Semi-Urban	13.8	14.2	12.9	
Rural	9.1	8.7	10.0	
Smoking Status (%)				
Smoking	23.1	23.5	22.1	0.740
Vaping	9.1	8.7	10.0	0.657
Smoking and Vaping	4.3	4.4	4.3	0.971
Smoking Cessation	11.8	10.5	15.0	0.160
Never Smoked	52.1	52.9	50.0	0.562

Bold signifies significant differences between sexes; BMI, body mass index; ^1^ refers to the sub-sample with active weight control efforts during the previous year (n = 367). Weight control methods are not mutually exclusive.

**Table 2:** Dietary intake and quality according to internalized weight bias tertile.

	WBIS Tertiles						
	Low, n = 165	Medium, n = 153	High, n = 166	*p*	*p*_Q1vsQ2_	*p*_Q2vsQ3_	*p*_Q1vsQ3_
WBIS-M (1–7)	**2.43 ± 0.38**	**3.40 ± 0.37**	**4.64 ± 0.65**	**<0.001**	**<0.001**	**<0.001**	**<0.001**
Sex (% females)	70.3	71.2	71.7	0.987			
Age (years)	**45.0 (26.0, 56.0)**	**37.0 (22.5, 51.0)**	**34.0 (22.8, 48.0)**	**<0.001**	**0.016**	0.584	**<0.001**
Maximum BMI (kg/m^2^)	**29.4 ± 4.2**	**31.4 ± 6.7**	**35.2 ± 8.4**	**<0.001**	**0.024**	**<0.001**	**<0.001**
Current BMI (kg/m^2^)	**27.4 ± 4.3**	**28.6 ± 5.6**	**33.0 ± 8.6**	**<0.001**	0.288	**<0.001**	**<0.001**
Overweight or Obesity (%)	**72.7**	**76.5**	**88.0**	**<0.001**	0.444	**0.008**	**<0.001**
Current to Max Weight (%)	95.8 (87.9, 100)	96.6 (89.3, 100)	95.9 (88.8, 100)	0.997			
Weight Control Efforts (in the previous year) (% yes)	**71.4**	**71.3**	**84.0**	**0.009**			
Energy Intake & Expenditure							
Energy Intake (kcal/24 h)	2027 (1598, 2809)	2053 (1633, 2568)	1986 (1589, 2812)	0.864			
Energy Intake (kcal/kg BW)	**26.4 (19.2, 36.9)**	**25.9 (19.6, 31.9)**	**23.8 (15.9, 32.0)**	**0.012**	0.112	0.987	**0.012**
Carbohydrates (% EI)	37.9 (33.4, 42.1)	37.6 (33.3, 42.5)	39.3 (34.8, 42.5)	0.277			
Protein (% EI)	17.4 (14.9, 19.4)	18.0 (15.8, 19.8)	16.7 (14.1, 20.1)	0.152			
Protein (g/kg BW)	**1.06 (0.83, 1.45)**	**1.10 (0.82, 1.43)**	**0.95 (0.70, 1.36)**	**0.033**	0.125	0.987	**0.046**
FAT (% EI)	43.2 (38.3, 48.1)	43.8 (39.0, 47.7)	42.5 (37.7, 47.3)	0.676			
MUFA (% EI)	21.3 (17.2, 24.3)	20.3 (17.1, 23.5)	20.3 (16.6, 22.8)	0.315			
SFA (% EI)	**11.8 (10.4, 13.6)**	**12.7 (11.1, 14.2)**	**12.3 (10.5, 14.0)**	**0.016**	**0.012**	0.625	0.099
Alcohol (% EI)	0.86 (0.17, 1.96)	0.76 (0.19, 1.55)	0.65 (0.00, 1.71)	0.253			
PAL	1.40 (1.33, 1.48)	1.41 (1.33, 1.50)	1.37 (1.31, 1.47)	0.155			
EI: EER (%)	88.2 (64.8, 119.6)	88.8 (65.1, 108.1)	81.4 (59.9, 116.7)	0.453			
Dietary Quality							
Non-refined Cereals (portions/d)	0.72 (0.16, 1.35)	0.50 (0.14, 1.02)	0.65 (0.24, 0.65)	0.064			
Vegetables (portions/d)	2.00 (1.23, 2.71)	1.52 (0.96, 2.27)	1.75 (0.97, 2.56)	**0.002**	**0.002**	0.119	**0.050**
Fruit (portions/d)	1.26 (0.63, 2.34)	1.06 (0.42, 2.13)	1.13 (0.49, 2.41)	0.401			
Olive Oil (portions/d)	1.0 (0.64, 1.0)	1.0 (0.64, 1.0)	1.0 (0.64, 1.0)	0.426			
Full-Fat Dairy (portions/d)	0.71 (0.28, 1.25)	0.85 (0.35, 1.28)	0.71 (0.28, 1.23)	0.276			
Potatoes (portions/w)	1.52 (0.83, 2.50)	1.47 (0.83, 2.16)	1.52 (0.83, 2.50)	0.818			
Legumes (portions/w)	3.68 (1.23, 3.68)	3.68 (1.23, 3.68)	3.68 (1.23, 3.68)	0.068			
Fish & Fisheries (portions/w)	3.68 (1.23, 4.90)	2.45 (1.23, 3.68)	2.45 (1.23, 3.68)	**0.017**	0.125	0.987	**0.018**
Poultry (portions/w)	3.68 (1.23, 3.68)	3.68 (2.45, 3.68)	3.68 (1.23, 3.68)	0.729			
Red Meat & Products (portions/w)	7.23 (4.17, 13.66)	9.31 (5.88, 15.32)	8.29 (4.48, 12.47)	0.066			
Alcohol (portions/w)	1.47 (0.49, 3.19)	1.47 (0.49, 2.45)	0.98 (0.00, 2.94)	0.725			
MedDietScore (0–55)	33.0 (29.0, 36.0)	31.0 (26.0, 34.0)	30.0 (25.8, 35.0)	**0.001**	**0.010**	0.987	**0.002**

Bold signifies significant differences between tertiles. WBIS-M, Modified Weight Bias Internalization Scale; BMI, body mass index; EI, energy intake; BW, body weight; PAL, Physical Activity Level; EER, Estimated Energy Requirement.

**Table 3:** Linear Regression Models exploring the relationship between dietary intake and quality and internalized weight bias (N = 484).

Linear Regression; Dependent Variable: Adherence to the Mediterranean Diet (0–55)				
	Model	B	95% CI	*p*
WBIS-M (per 1 unit)	Crude	**−1.059**	**−1.566, −0.553**	**<0.001**
	Adjusted ^1^	**−0.629**	**−1.215, −0.044**	**0.035**
	Fully Adjusted ^2^	**−0.670**	**−1.257, −0.083**	**0.025**
**Linear Regression; Dependent Variable: Energy Intake (per 1 kcal/kg)**				
WBIS-M (per 1 unit)	Crude	**−1.560**	**−2.624, −0.496**	**0.004**
	Adjusted ^1^	−0.290	−1.441, 0.861	0.621
	Fully Adjusted ^2^	−0.219	−0.484, 0.047	0.107
**Linear Regression; Dependent Variable: Protein Intake (per 1 g/kg)**				
WBIS-M (per 1 unit)	Crude	**−0.083**	**−0.134, −0.032**	**0.002**
	Adjusted ^1^	−0.021	−0.077, 0.036	0.474
	Fully Adjusted ^2^	−0.015	−0.048, 0.017	0.348
**Linear Regression; Dependent Variable: Saturated Fat Intake (per 1% of Energy Intake)**				
WBIS-M (per 1 unit)	Crude	**0.253**	**0.032, 0.474**	**0.025**
	Adjusted ^1^	0.085	−0.175, 0.344	0.522
	Fully Adjusted ^2^	**0.258**	**0.021, 0.496**	**0.033**

^1^ Adjusted for age, sex (woman vs. man), current BMI; ^2^ adjusted for age, sex (woman vs. man), current BMI, financial status (having income equal or higher than expenses or not), family status (being married/in a relationship or not), EI:EER, weight control efforts (yes or no); WBIS-M, Weight Bias Internalization Scale; BMI, body mass index; EI, energy intake; EER, Estimated Energy Requirement.

**Table 4:** Multinomial Logistic Regression Models exploring the relationship between dietary intake and quality and internalized weight bias (N = 484).

Odds Ratio (95% CI) of Being Classified in the Medium or Highest WBIS-M Tertile (by Sex)				
	Model	Lowest	Medium	Highest
MedDietScore (per 1 unit)	Crude	1.000 (reference)	**0.940 (0.903, 0.978)**	**0.929 (0.894, 0.966)**
	Adjusted ^1^	1.000 (reference)	**0.948 (0.910, 0.988)**	**0.946 (0.908, 0.986)**
	Fully Adjusted ^2^	1.000 (reference)	**0.950 (0.910, 0.993)**	**0.942 (0.901, 0.984)**
Energy Intake (per 1 kcal/kg)	Crude	1.000 (reference)	**0.982 (0.964, 1.000)**	**0.974 (0.956, 0.992)**
	Adjusted ^1^	1.000 (reference)	0.983 (0.964, 1.001)	**0.980 (0.961, 1.000)**
	Fully Adjusted ^2^	1.000 (reference)	0.962 (0.888, 1.041)	0.805 (0.737, 0.878)
Protein Intake (per 1 g/kg)	Crude	1.000 (reference)	0.796 (0.554, 1.144)	**0.549 (0.366, 0.823)**
	Adjusted ^1^	1.000 (reference)	0.800 (0.547, 1.172)	**0.576 (0.369, 0.899)**
	Fully Adjusted ^2^	1.000 (reference)	1.257 (0.621, 2.546)	**0.280 (0.125, 0.630)**
SFA (per 1% of EI)	Crude	1.000 (reference)	**1.115 (1.019, 1.220)**	1.071 (0.982, 1.169)
	Adjusted ^1^	1.000 (reference)	**1.111 (1.015, 1.217)**	1.056 (0.964, 1.157)
	Fully Adjusted ^2^	1.000 (reference)	**1.110 (1.011, 1.219)**	1.069 (0.972, 1.176)

^1^ Adjusted for age, having BMI ≥ 25 kg/m^2^ (yes or no); ^2^ adjusted for age having BMI ≥ 25 kg/m^2^ (yes or no), financial status (having income equal or higher than expenses or not), family status (being married/in a relationship or not), EI:EER, weight control efforts (yes or no); WBIS-M, Weight Bias Internalization Scale; SFA, saturated fatty acid; BMI, body mass index; EI, energy intake; EER, Estimated Energy Requirement.

## Data Availability

Data may be available upon reasonable request submitted to the corresponding author (D.P.), due to restrictions related to legal or ethical reasons.

## Abbreviations

The following abbreviations are used in this manuscript:

MeDi	Mediterranean Diet
WBIS-M	Weight Bias Internalization Scale—Modified
BMI	Body Mass Index
FFQ	Food Frequency Questionnaire
EI	Energy Intake
IPAQ-sf	International Physical Activity Questionnaire—short form
MET	Metabolic Equivalent of Task
PAL	Physical Activity Level
EER	Estimated Energy Requirements
SFA	Saturated Fatty Acid
